# Emphysematous osteomyelitis

**DOI:** 10.1590/0037-8682-0291-2024

**Published:** 2025-01-27

**Authors:** Paula Castro, Miriam Menna Barreto, Edson Marchiori, Rosana Souza Rodrigues

**Affiliations:** 1 Universidade Federal do Rio de Janeiro, Rio de Janeiro, RJ, Brasil.; 2 Instituto D'Or de Pesquisa e Ensino, Rio de Janeiro, RJ, Brasil.

A 65-year-old man with diabetes and paraplegia, due to spinal cord trauma sustained approximately 30 years ago, presented with a chronic pressure ulcer in the sacral region. He was admitted with fever and discharge from the ulcer and was diagnosed with sepsis. He had previously been treated with an antibiotic regimen, but his symptoms did not improve. Laboratory tests revealed elevated inflammatory markers, including leukocytosis and increased C-reactive protein levels. Antibiotic treatment with ampicillin and sulbactam was initiated.

**FIGURE 1:**
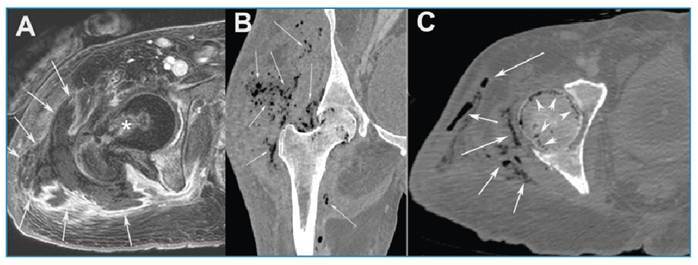
A fat-suppressed T1-weighted MR image acquired after intravenous contrast administration **(A)** reveals inflammatory changes in the periarticular soft tissues and abscessed areas (arrows). The bone marrow of the proximal femur and acetabulum shows mild enhancement (asterisk). Coronal **(B)** and axial **(C)** non-enhanced CT images (bone window) demonstrating multiple small foci of intramedullary gas in the right femoral head and neck (arrowheads), along with soft-tissue emphysema (arrows).

Magnetic resonance imaging of the pelvis revealed an extensive soft tissue collection with gas foci in the right trochanteric region, as well as intraosseous gas. A purulent collection was drained from the right thigh (Figure 1A). The culture showed growth of *Klebsiella pneumoniae*, prompting the escalation of antibiotic treatment to ciprofloxacin.

A computed tomography scan of the abdomen, performed one week later, revealed collections and gas foci in the right groin and thigh, as well as gas foci in the right femur and acetabulum, with bone erosion (Figure 1B, C). Another drainage procedure was performed, evacuating a large amount of purulent secretions. The patient is currently in the intensive care unit, showing progressive clinical improvement.

Emphysematous osteomyelitis is a rare and severe form of bone infection, characterized by the presence of gas within bone tissue owing to bacterial activity. Enterobacteriaceae and anaerobes are the most common causative agents. Most cases occur in patients with diabetes. Early detection and appropriate treatment are essential to prevent serious complications and improve the prognosis^1^ ^-^ ^4^.
